# Pathological Methods Applied to the Investigation of Causes of Death in Developing Countries: Minimally Invasive Autopsy Approach

**DOI:** 10.1371/journal.pone.0132057

**Published:** 2015-06-30

**Authors:** Paola Castillo, Esperança Ussene, Mamudo R. Ismail, Dercio Jordao, Lucilia Lovane, Carla Carrilho, Cesaltina Lorenzoni, Marcus V. Lacerda, Antonio Palhares, Leonardo Rodríguez-Carunchio, Miguel J. Martínez, Jordi Vila, Quique Bassat, Clara Menéndez, Jaume Ordi

**Affiliations:** 1 ISGlobal, Barcelona Ctr. Int. Health Res. (CRESIB), Hospital Clínic-Universitat de Barcelona, Barcelona, Spain; 2 Department of Pathology, Maputo Central Hospital, Maputo, Mozambique; 3 Fundação de Medicina Tropical Dr. Heitor Viera Dourado, Manaus, Amazonas, Brazil; 4 Centro de Investigação em Saúde de Manhiça, (CISM), Maputo, Mozambique; 5 Department of Microbiology, Hospital Clinic, Universitat de Barcelona, Spain; 6 Department of Pathology, Hospital Clinic, Universitat de Barcelona, Spain; University of Palermo, ITALY

## Abstract

**Background and Aims:**

Complete diagnostic autopsies (CDA) remain the gold standard in the determination of cause of death (CoD). However, performing CDAs in developing countries is challenging due to limited facilities and human resources, and poor acceptability. We aimed to develop and test a simplified minimally invasive autopsy (MIA) procedure involving organ-directed sampling with microbiology and pathology analyses implementable by trained technicians in low- income settings.

**Methods:**

A standardized scheme for the MIA has been developed and tested in a series of 30 autopsies performed at the Maputo Central Hospital, Mozambique. The procedure involves the collection of 20 mL of blood and cerebrospinal fluid (CSF) and puncture of liver, lungs, heart, spleen, kidneys, bone marrow and brain in all cases plus uterus in women of childbearing age, using biopsy needles.

**Results:**

The sampling success ranged from 67% for the kidney to 100% for blood, CSF, lung, liver and brain. The amount of tissue obtained in the procedure varied from less than 10 mm2 for the lung, spleen and kidney, to over 35 mm2 for the liver and brain. A CoD was identified in the histological and/or the microbiological analysis in 83% of the MIAs.

**Conclusions:**

A simplified MIA technique allows obtaining adequate material from body fluids and major organs leading to accurate diagnoses. This procedure could improve the determination of CoD in developing countries.

## Introduction

Complete diagnostic autopsy (CDA) is considered the gold standard methodology to inform on cause of death (CoD)[1]. Even in the era of high-tech medicine, major clinico-pathological discrepancies are reported in up to a quarter (23.5%) of CDAs[2].

In developing countries non-forensic CDA has always been an infrequent procedure due to several reasons. First of all, most deaths occur outside the health system, thus precluding not only the post-mortem evaluation but frequently even basic medical assistance[3,4]. Secondly, the limited human resources, particularly of trained personnel with technical expertise in this procedure, are a major limitation to undertake CDAs even in tertiary or reference hospitals. Finally, problems related to cultural and/or religious acceptance may negatively influence consent and, consequently, the practice of CDAs in some regions.

To overcome this problem the WHO recommends verbal autopsy (VA), a structured interview to family, friends, and caretakers, as an alternative to CDA in developing countries[5–8]. It has been shown that VA provides mainly a broad syndromic approach, but its performance for specific etiologic diagnosis is poor. Moreover, VA may misclassify a substantial number of deaths, and different researchers using this tool may obtain different results. Recently published estimates on various causes of global cause-specific mortality[9,10] have stirred a profound debate about the validity and adequacy of VA to estimate CoD[5].

Minimally invasive autopsy (MIA) has recently been proposed as an alternative to CDA[11–16]. At present, MIA most often includes the use of imaging techniques, such as MRI or CT scan, coupled with targeted small diagnostic biopsies of key organs obtained by needle puncture[11]. The results obtained so far with this technique seem to be reliable and comparable to the CDA, particularly in stillbirths and neonatal deaths[17–19]. However, these current MIA protocols are neither adequate nor feasible in developing countries due to the need of high-tech imaging studies that, if available, cannot be used for post-mortem studies. Although some studies have performed post mortem biopsies they are often limited to a single organ[12,15,16]. Thus, there is a need for simplified, standardized MIA protocols adequate for low-income settings that can provide accurate information on CoD. We aimed to develop and test a MIA procedure involving organ-directed sampling with pathological and microbiological analyses feasible for trained technicians in low-income settings.

## Materials and Methods

### Study Design

As part of an ongoing validation study of the MIA compared with the CDA (CaDMIA project) [20] a standard operating procedure (SOP) for the MIA tool was initially developed at the Hospital Clinic of Barcelona (HCB), and later tested and modified at the Department of Pathology of the Maputo Central Hospital (MCH). The study protocol was approved by the National Mozambican Ethics Committee (ref. 342/CNBS/13) and the Ethics Committee of the HCB. For all of the autopsies, a CDA had been requested by their clinicians as part of the medical evaluation and the family had given an informed verbal consent to perform the study.

### The MIA procedure

The MIA procedure was performed by a single pathologist with the assistance of a technician. It begins by evaluating the liver, spleen, kidneys and other abdominal organs with a portable ultrasound (US) scan device (Mindray Z6, Mindray Med Int Ltd, Shenzhen, China). In women of childbearing age a suprapubic US scan of the pelvis is also performed. All the lesions identified, abnormal fluids (ascites, pleural effusions), as well as the position of deep organs (particularly the spleen and the kidneys), are recorded. Then, the US gel is removed with cellulose paper and the areas of the corpse to be punctured are cleaned and sterilized initially with tap water and then with 96° alcohol and iodine solution, allowing 5 minutes for each act on the surface of the corpse in order to achieve adequate disinfection for the microbiological analyses[21]. During the cleaning process the skin is carefully inspected in search of any macroscopically evident lesion.

**Fig 1:**
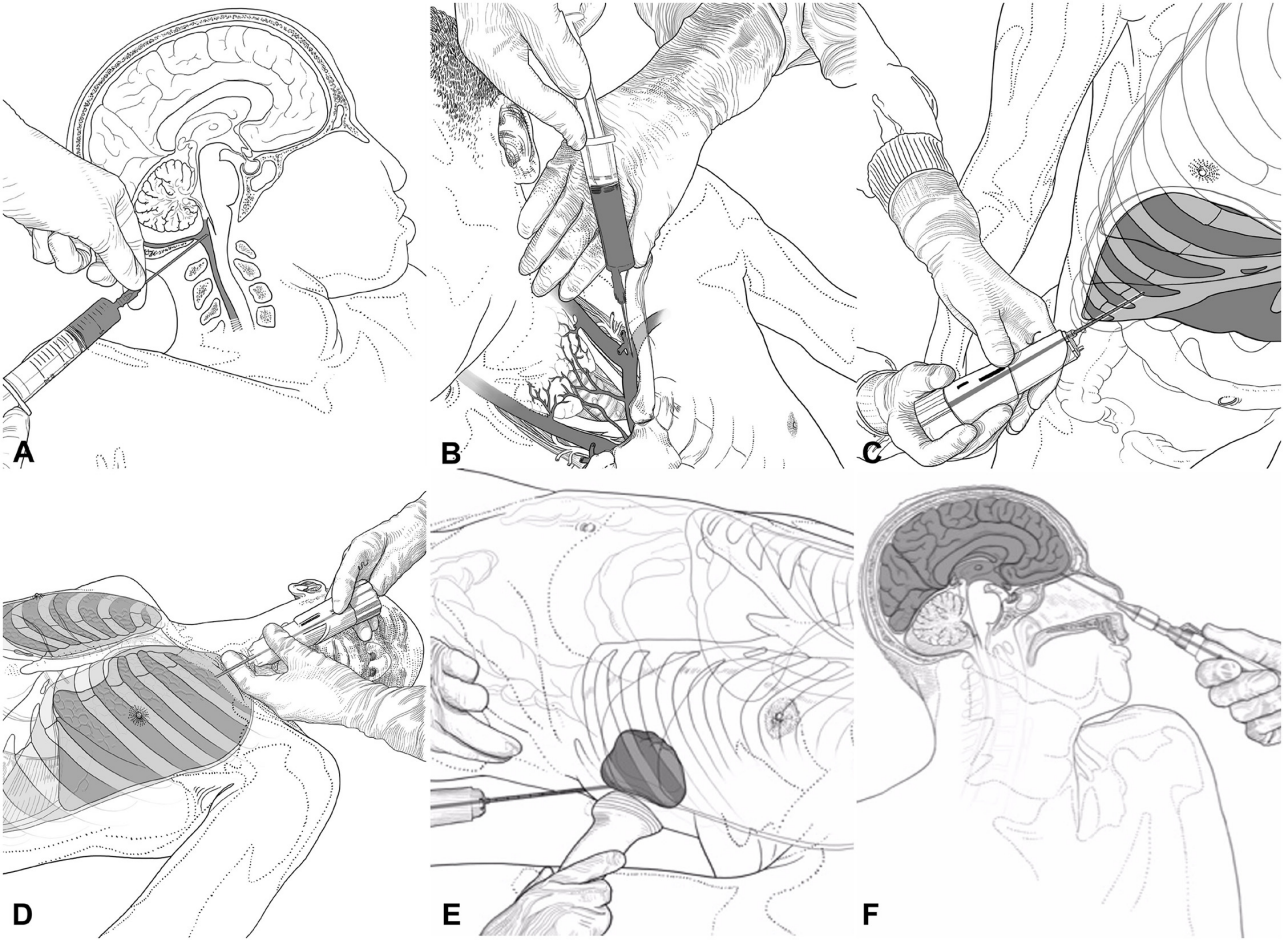
Procedures for the collection of cerebrospinal fluid (A), peripheral blood (B), liver (C), lung (D), spleen (E), and the central nervous system biopsy (F) (designed by Xabier Sagasta).

The type and main characteristics of the different needles used in the MIA procedure for each particular biopsy, the sites of puncture and the number of samples to be obtained are summarized in Table 1 and illustrated in Fig 1. The sampling process starts with the collection of CSF by occipital puncture, reaching the *cisterna magna* (Fig 1A). Up to 5 mL of CSF are collected in a 10 mL sterile tube and two aliquots of 2 mL each are stored in Eppendorf Safe-Lock Tubes at -80°C. Then, 20 mL of blood are extracted by puncture of the subclavian vein through a supraclavicular or infraclavicular approach (Fig 1B). If less than 20 mL of blood are obtained with this approach, intracardiac blood is targeted through a heart puncture at the left 5^th^ intercostal space. Ten mL of blood are collected in an EDTA-containing tube, 10 mL in an aerobic adult or pediatric blood culture bottle (BACTEC), and four large drops of blood are placed onto a Whatman 903 filter (GE Healthcare Bio-Sciences, Pittsburgh, PA, USA). If other fluids (pleural effusion or ascites) are detected in the US scan, 20 mL are collected. After the body fluids have been collected, specimens of the major organs are taken.

**Table 1:** Type and main characteristics of the different needles used in the minimally invasive autopsy procedure for each particular biopsy, puncture sites and number of samples to be obtained. The organ tissues are presented in the order in which the samples were collected.

Tissue	Needle	Type	Gauge	Needle length (mm)	Puncture site	Volume/Number of samples for microbiology	Number of samples for histology
Cerebrospinal fluid (CSF)	Quincke Spinal ^#^	Manual	20	100	Occipital puncture	20 mL	-
Blood	Quincke Spinal	Manual	20	100	Supra/infra-clavicular or left ventricle	20 mL	-
Liver	Unicut *	Manual	14	115	Anterior right axillar line, 11th-12th intercostal space	2 cylinders	4–6 cylinders
Lungs	Monopty*	Automatic	14	100	Right and left clavicular region down to the diaphragm for microbiology samples. Multiple random thoracic punctures for pathology	2 from left lung, 2 from right lung	4–6 cylinders from each side
Heart	Monopty*	Automatic	14	100	Left thoracic region 5th intercostal space in a parasternal point	-	2 cylinders
Spleen	Monopty*	Automatic	14	160	Posterior left axillar line in the 11th-12th intercostal space (locate with US scan)	-	2 cylinders
Kidneys	Monopty*	Automatic	14	160	Upper abdominal/lumbar area (locate with US scan)	-	2 cylinders
Bone Marrow	T-LokTrephine**	Manual	8	100	Anterior iliac crest	Half of the cylinder	Half of the cylinder
CNS	Biomol***	Semi Automatic	16	200	Trans-ethmoidal puncture. Perforation of the cribriform plate with the bone marrow trephine to reach the cranial cavity	2 cylinders	4–8 cylinders
Uterus	Monopty*	Automatic	14	160	Central suprapubic region (locate with US scan)	-	2 cylinders
Skin	Biopsy punch ^##^	Manual	-	5	Macroscopically detected lesions	-	2–3 biopsy punches

^#^ Becton Dickinson, FranklinLakes, NJ, USA

^##^KAI Europe GMBH, Solingen, Germany

* BARD Biopsy Systems, Tempe, AZ; USA

**Mana-Tech Ltd, Staffordshire, UK

*** HS Hospital Service S.P.A, Rome, Italy

### Evaluation of the procedure

The evaluation of the procedure was conducted at the MCH, a government-funded quaternary health care facility that serves as the referral center for other hospitals in Southern Mozambique. Mozambique has a total population of around 25, 2 million and the life expectancy at birth in both sexes is 52/53 years[22]. In terms of the number of years of life lost (YLLs) due to premature death in Mozambique, HIV/AIDS, malaria, and lower respiratory infections were the highest ranking causes in 2010 [23].

This study was performed from November 4 to 23, 2013. The initial SOP was applied in a group of 30 adults who died at the MCH. Of all the CDAs requested every day at the Department of Pathology of the HCM (between 5 and 12) two autopsies per day were selected on the basis of most recent time of death. All the deceases had occurred less than 24 hours prior to the procedure A CDA had been requested by the clinicians for all these cases following the standard procedures of the hospital, and the families gave verbal informed consent for the procedure.

The first samples from the liver, right and left lung and CNS biopsies were taken using a sterile new needle, and placed into thioglicolate broth (first sample) and lysis buffer (second sample) for microbiology studies. The subsequent samples were taken using the same needles, and the material obtained was fixed inside a cassette for histology. Only one biopsy of the bone marrow was obtained, and this was divided into two samples, one for microbiology and the other for histological evaluation.

In women of childbearing age a sample from the uterus was taken by central suprapubic puncture. If amniotic fluid was detected, 20 mL was collected in a sterile tube. Finally, if skin lesions were observed a biopsy using a punch was performed at the border of the grossly identified lesion avoiding disfiguration of visible body areas.

The time spent to complete the procedure was recorded for each case.

### Histological laboratory procedure

Tissue specimens for histological analysis were fixed in 10% neutral buffered formalin for 4 hours, passed into distilled water, embedded in paraffin, and cut into four-micron sections, which were then stained with H&E as per standard procedures.

### Microscopy and analysis of the tissue samples for histological analysis

All samples taken during the MIA procedure were evaluated using an Olympus BX51 light microscope. H&E sections of all the tissues were initially evaluated. When necessary, ancillary histochemical (i.e. Zieh-Neelsen, PAS, Grocott stain, etc.) and immunohistochemical stains were performed in selected blocks to confirm or exclude specific lesions suspected on the H&E stains. Immunohistochemical staining for CD45 (leukocyte common antigen) was performed in all cases in the CNS samples in order to exclude/confirm minimal leukocyte infiltrates.

All the cylinders present in each block were carefully evaluated, recording all the organs present and measuring the area (in mm^2^) of each organ obtained in the MIA procedure after drawing the perimeter of each tissue cylinder in a Leica dmd 108 microscope.

### Determination of the putative cause of death

The histological evaluation of the MIA samples was performed by two pathologists who were blinded to the clinical data or the results of the CDA. The microbiological analyses were supervised by an experienced microbiologist and included conventional blood cultures, CSF, the CNS, liver and lungs, testing for antibodies against hepatitis B and C viruses and HIV and PCR analysis for Plasmodium falciparum and Mycobacterium tuberculosis in all the cases. These routine analyses were complemented by specific molecular assays guided by the results of the histological evaluation and the conventional cultures. A detailed description of the microbiological analyses has been published elsewhere. The results obtained independently in the histological and the microbiological analyses were discussed in a joint session composed by the pathologists, the microbiologist, and an epidemiologist as well as a clinician with expertise in infectious and tropical diseases, and a final putative CoD was assigned to each case based on the combination of all the findings obtained with the MIA samples.

## Results

### Efficiency of the sampling procedure

**Fig 2:**
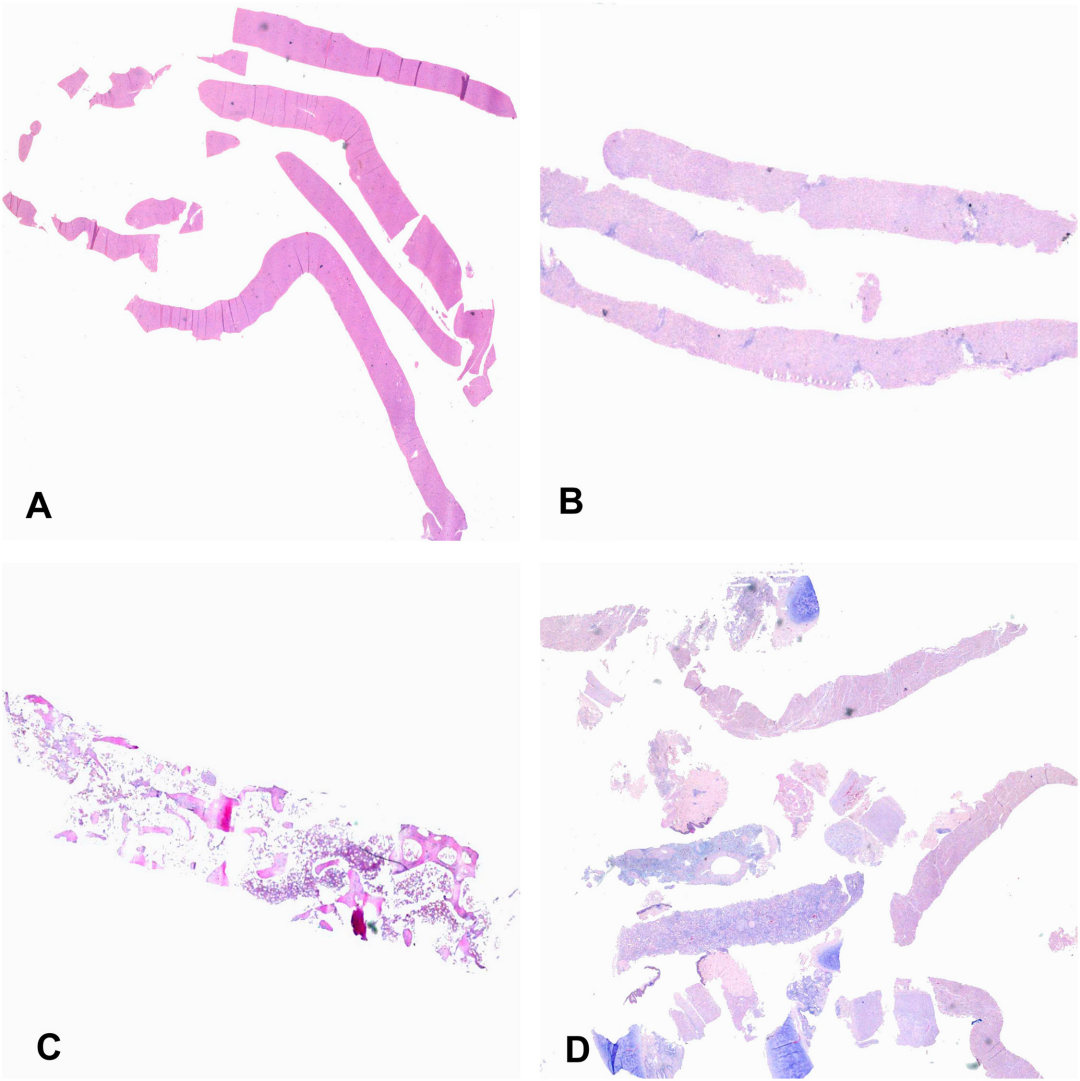
Representative image of the samples obtained from the central nervous system (A), liver (B), bone marrow (C) and lungs/heart and kidney (D) in the minimally invasive autopsy procedure.

**Fig 3:**
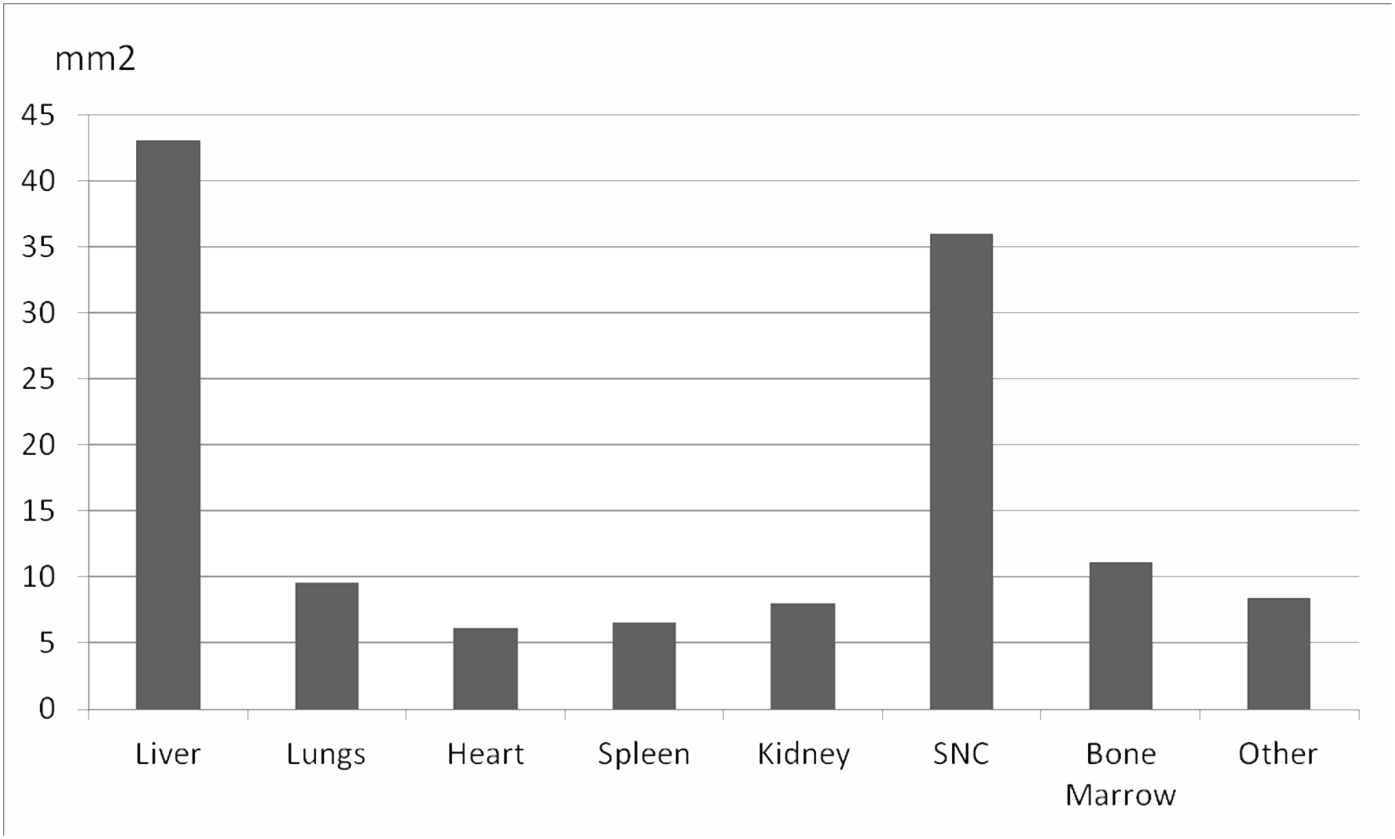
Median area of tissue for histological evaluation obtained from each organ in mm^2^.

In all the cases, adequate samples of blood and CSF were collected. The percentage of success in terms of adequate samples for histological evaluation was 66.7% (20/30) for the kidney, 70% (21/30) for the spleen, 80% (24/30) for the heart, 96.7% (29/30) for the bone marrow and 100% (30/30) for the liver, lungs and CNS. The skin was sampled only when gross lesions were macroscopically identified (9/30 cases). The uterus was sampled in 11/14 women of childbearing age. In only 54% (6/11) of these cases uterine tissue was successfully obtained for histological evaluation. The number of cylinders obtained from each organ ranged from 0–4 for the kidneys, heart and spleen, 1–4 for the lungs, and 7–9 for the liver and CNS. A representative image of the samples obtained from the CNS, liver, bone marrow and lungs/heart and kidney is shown in Fig 2. Fig 3 shows the median area of tissue sampled in each organ per case. The median area of tissue obtained ranged from 42 mm^2^ for the liver, to less than 10 mm^2^ for organs such as the lungs and the heart.

A learning curve was observed in terms of time to complete the entire MIA procedure by the pathologist, reducing from 90 minutes for the first procedure performed to 30–45 minutes from the third procedure and thereafter.

### Characteristics of the autopsies and diagnostic performance of the MIA

The MIA analysis of 30 cases included 18 women (60%) and 12 males (40%) with a median age of t 35 years (range 17–76). Nineteen out of the 30 (63%) patients were HIV positive.

**Fig 4:**
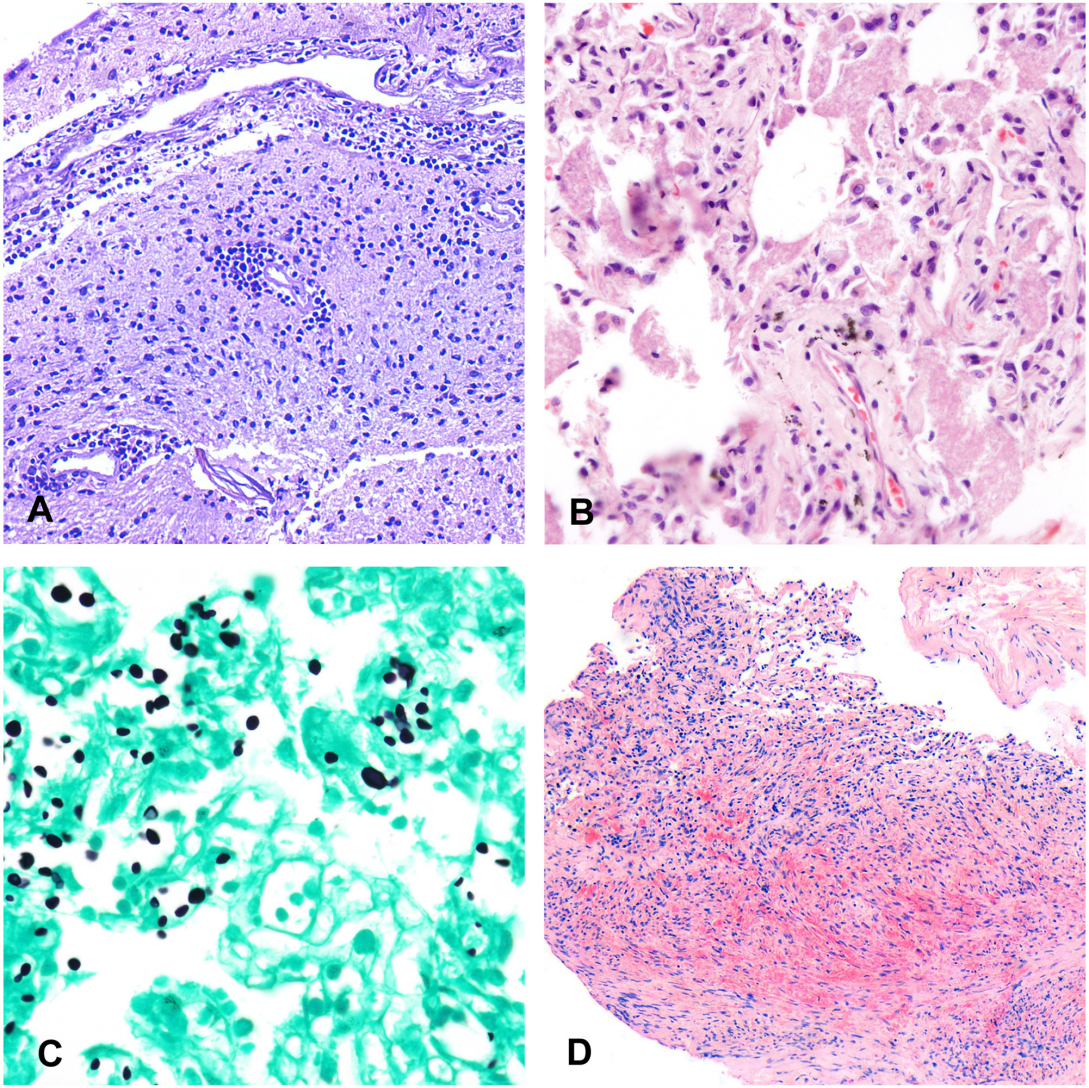
Representative examples of putative causes of death identified with minimally invasive autopsy sampling. A) Meningoencephalitis (hematoxylin and eosin, 200x); B) *Pneumocysttis jiroveci* pneumonia (hematoxylin and eosin, 200x); C) *Cryptococcus neoformans* infecting the lung (PAS metenamine silver stain, 200x); D) Kaposi’s sarcoma involving the lung (hematoxylin and eosin, 100x).

Table 2 shows the basic demographic data of the patients included in the study, the HIV status, the diagnoses reached in the histological evaluation of the samples obtained through the MIA, the results of the microbiological analyses and the final diagnoses obtained after combining the pathological and the microbiological results. A putative CoD was identified in the histological and/or the microbiological analysis in 83% of the MIAs. A severe disease considered as a possible CoD was identified in the histology samples in 22/30 cases (73%), with 14 cases being infectious diseases, and 8 non- communicable diseases including 4 malignant tumors. Three of the malignant tumors were of viral origin (two liver cell carcinomas, 1 Kaposi’s sarcoma) and one was a large B cell lymphoma negative for the *in situ* hybridization for Epstein- Barr virus. The MIA tended to yield a CoD in patients 35-year-old or younger more frequently than in those older than 35 years of age, although the difference was not statistically significant (12/17; 70.6% vs. 7/14, 50%; p = 0.288). Fig 4 shows representative examples of CoD identified through MIA sampling.

**Table 2:** Age, sex, HIV status and pathological and microbiological diagnoses obtained in the 30 minimally invasive autopsies (MIA) performed in the pilot study.

MIA	Age	Sex	HIV status	Pathological diagnosis	Microbiological diagnosis	Final diagnosis
1	27	M	-	Pyogenic-granulomatous meningoencephalitis	Rhizopus oryzae	*R*. *oryzae*cerebral invasive mucormicosis
2	76	M	-	Cerebral infarction *	-	Cerebral infarction
3	32	M	-	Hepatocellular carcinoma	HBV	Hepatocellular carcinoma (HBV)
4	19	F	-	Non specific	-	Non specific
5	25	F	-	Cardiac hyperthrophy	-	Suggestive of cardiovascular disease
6	28	F	+	Disseminated Kaposi’s sarcoma ^#^	HHV-8 ^#^	Disseminated Kaposi’s sarcoma HHV-8
7	35	F	+	Pneumocystis pneumonia ^#^	*Pneumocystis jiroveci*	Pneumocystis pneumonia
8	49	M	-	Cardiac hyperthrophy	-	Suggestive of cardiovascular disease
9	35	F	+	Disseminated necrotizing granulomas	*Toxoplasma gondii*	Cerebral toxoplasmosis
10	27	F	+	Pyogenic pneumonia	*Acinetobacter baumannii*	*A*. *baumannii* sepsis of pulmonary origin
11	33	M	+	Meningoencephalitis **	*Streptococcus pneumoniae*	Septic pneumococcal meningitis
12	41	M	+	Pyogenic meningoencephalitis	Cytomegalovirus	Pyogenic meningoencephalitis
13	76	F	-	Non specific	-	Non specific
14	17	F	+	Pyogenic meningoencephalitis	*Mycobacterium tuberculosis*	Tuberculous meningitis
15	43	M	+	Pyogenic pneumonia	*Pneumocystis jiroveci*	Pneumocystis pneumonia
16	48	M	-	Hepatocellular carcinoma	Hepatitis B virus	Hepatocellular carcinoma (HBV)
17	53	F	+	Large B cell lymphoma	-	Large B cell lymphoma
18	36	F	+	Non specific	-	Non specific
19	62	M	-	Cardiac hypertrophy	-	Suggestive of cardiovascular disease
20	35	M	+	Pyogenic pneumonia	-	Pyogenic pneumonia
21	29	F	+	Disseminated necrotizing granulomas ^#^	*Mycobacterium tuberculosis*	Miliary tuberculosis
22	61	F	-	Meningoencephalitis **	-	Suggestive of meningoencephalitis
23	30	F	+	Pyogenic meningoencephalitis	*Cryptococcus neoformans*	Cryptococcal meningitis
24	74	M	-	Non specific	-	Non specific
25	57	F	+	Non specific	-	Non specific
26	45	F	+	Disseminated necrotizing granulomas ^#^	*Mycobacterium tuberculosis*	Miliary tuberculosis
27	27	F	+	Non specific	*Enterococcus faecalis*, *Mycoplasma hominis*	Puerperal sepsis
28	29	F	+	Cryptococcal sepsis ^#^	*Cryptococcus neoformans*	Cryptococcal sepsis
29	30	M	+	Non specific	*Streptococcus dysgalactiae*	*S*. *dysgalactiae* septicemia
30	31	F	+	Non specific	*Toxoplasma gondii*	Cerebral toxoplasmosis

M: male; F: female

* necrosis and hemorrhage in the cerebral parenchyma with negative stains for microorganisms

^#^ etiological agent detected in the pathology sample by immunohistochemistry or special stains

HBV: hepatitis B virus

HHV-8: human herpes virus 8

** minimal perivascular inflammatory infiltrate in CNS parenchyma after immunohistochemical analysis against CD45.

Significant diagnostic information for CoD determination was obtained from the histological samples from the lung, CNS and liver (6 cases each), spleen (3 cases) and skin (1 case). In contrast, the samples from bone marrow, heart, kidney or uterus rendered no information on CoD. For the microbiological samples, the diagnostic information was obtained from the CNS (12 cases), CSF (9), lung (8), liver (7) and blood (3). The microorganism was identified in a single organ in 4 cases, in two organs in 7, in three organs in 1 and in four and five organs in two each.

Meningoencephalitis was the leading CoD (6 cases) among the infectious diseases. The histological samples of the CNS showed clear pyogenic and/or granulomatous reaction in four cases and only a mild perivascular inflammatory infiltrate, enhanced in the immunohistochemical analysis with CD45, in the remaining two cases. In 5/6 of the meningoencephalitis an increase in the leukocyte count was identified in the CSF. The etiologic agent causing the death was identified only by histological techniques in one case (human herpes virus 8 in Kaposi’s sarcoma), by both special histological stains and microbiological techniques in 4 cases, and only by microbiological analyses in 10 cases. In 6 out of these 10 cases a pathological lesion attributable to the microorganism identified was clearly observed, whereas in three cases the final infectious CoD was achieved only by microbiological analyses in the context of non-specific histological changes. In these cases the microorganisms putatively causing the death were identified in multiple organs and fluids (*M*. *hominis* and *E*. *faecalis* [case 27], *S*. *dysgalactiae* [case 29]) or in both the brain and the CSF (*T*. *gondii*, *case 30*).

## Discussion

There is a need to find alternative post-mortem examination methods that can be used as tools to advance medical knowledge on CoD and thereby guide effective interventions in low-income countries. This study has developed a standardized protocol of MIA for low-income settings, which has shown to provide adequate samples for both histological and microbiological analysis from most major organs. This simplified technique can be implemented in developing countries and would allow the etiologic diagnosis of a significant number of patients. This standardized protocol of MIA developed achieved the major aims of the study allowing: 1 the collection of as many tissue samples as necessary to make a reliable final diagnosis; and 2) the tissue samples to be adequately preserved in order to maintain high quality material for the analyses. The decision as to the organs to be sampled was taken according to previous experience in autopsy studies[24–27]. During the preparation of the standard operating procedure the observation that the spleen and the kidney were difficult to locate led us to introduce the use of US scan to identify these organs. Once the organ was located, the puncture was done without US guidance. The selection of the type and gauge of the needles to be used was based on clinical experience as well as in the scant information reported in other postmortem studies[4]. The thickest available needles were selected because of the impossibility of clinical complications.

In this study the percentage of success was 100% for the liver, lung and CNS and slightly lower for the other organs. These findings are concordant with other studies [18,28] that, although including a small number of cases, also reported successful sampling of organs such as the lung. The current findings were also similar to those reported in a study carried out almost 45 years ago that included 294 adult cases, in which adequate samples were obtained from the liver in 92% of cases, the heart in 55%, the lung in 46% and the kidney in 34% [28]. The reduced percentage of success for the sampling of the uterus in women of childbearing age, an organ not targeted in previous studies, could be explained by its difficult accessibility behind the urinary bladder. Few previous studies have attempted to collect CNS samples in autopsies[15,29]. In two of the studies performed in children [12,15] a supraorbital puncture was used. In a study from Uganda a trans-nasal approach was used, as in our series, because the supraorbital plate in adults is completely ossified and extremely resistant to perforation, whereas the cribriform plate is softer and easier to pass through with a bone trephine. The percentage of success in CNS sampling in our study (100%) was identical to the success rate obtained in the Malawian study [12] and very similar to that obtained in the Ugandan study [30]. Finally, although the rate of success in sampling attainment was high for many organs, the median area of tissue sampled in each organ was quite different; suggesting that the small size of the samples obtained for several organs such as the lung, heart, spleen and kidney may hinder reaching a diagnosis in focal lesions.

In this study a severe disease considered as a possible CoD was identified in the histological analysis in more than half of the cases (60%). The leading CoD was an infectious disease. This diagnosis tended to be more frequent among 35-year-old or younger patients than in those older than 35. This is consistent with infectious diseases being more frequent in young compared to older patients at least in developing countries[31]. Over 60% of the patients had uncontrolled or poorly controlled HIV infections. The highly widespread infections occurring in these immunocompromised hosts may have positively impacted the ability to detect the disease and the pathogen in the limited samples obtained in the MIA.

Importantly, significant diagnostic information for CoD determination was obtained from the samples of the lung, the CNS, liver, spleen and skin, all of which are organs frequently involved in infectious diseases, whereas the samples from bone marrow, heart, kidney and uterus rendered little or no information on CoD. If these findings are confirmed in subsequent studies, the MIA procedure could be significantly simplified by eliminating the use of US examination and the sampling of these non-contributive organs.

Interestingly, in this study it was possible to obtain adequate material for microbiological analysis, which allowed confirmation of the pathological results and the etiological characterization of the microorganisms causing the death in a significant number of cases, including fungal, viral and bacterial diseases. In one case (case 12) the pathological exam identified a meningoencephalitis and cytomegalovirus was detected by microbiological methods. Although meningoencephalitis can rarely [32] be associated with cytomegalovirus, in the absence of cytomegalic inclusions in the tissue, the disease cannot be conclusively attributed to this infection. The main limitations of the MIA procedure are related to the cost of the biopsy needles, the requirement of complex and expensive microbiological platforms and qualified microbiologists, and the need for pathologists specifically trained in the evaluation of small samples with limited representation of the lesions. Another possible limitation is that the diagnostic success of the procedure may be influenced by the dissemination of the disease, and could be significantly reduced in focal lesions and in limited infections in immunocompetent hosts. Finally, whereas MIA provided a high number of infectious diseases putatively causing the death, it is likely to be less effective for non-infectious diseases.

In conclusion, the results of this study confirm that MIA is a feasible and reliable tool that allows successful collection of samples from key organs. Further studies comparing the results of MIA with those of CDA are ongoing to test the validity of MIA. This procedure could be a valuable tool to improve the knowledge of CoD in developing countries where infectious diseases are common causes of death.
